# Informed consent in randomised controlled trials: development and preliminary evaluation of a measure of Participatory and Informed Consent (PIC)

**DOI:** 10.1186/s13063-017-2048-7

**Published:** 2017-07-17

**Authors:** Julia Wade, Daisy Elliott, Kerry N. L. Avery, Daisy Gaunt, Grace J. Young, Rebecca Barnes, Sangeetha Paramasivan, W Bruce Campbell, Jane M. Blazeby, Alison J Birtle, Rob C. Stein, David J Beard, Alison W Halliday, Jenny L. Donovan, On behalf of the ProtecT study group, CLASS study group, Chemorad study group, POUT study group, OPTIMA prelim study group, CSAW study group and ACST-2 study group

**Affiliations:** 1 School of Social and Community Medicine, University of Bristol, 39 Whatley Road, Clifton, Bristol, BS8 2PS UK; 2 Royal Devon and Exeter Hospital, Exeter, EX2 5DW UK; 3 Rosemere Cancer Centre, Royal Preston Hospital, Sharoe Green Lane North, Fulwood, Preston, Lancashire, PR2 9HT4 UK; 4 University of Manchester, Oxford Road, Manchester, M13 9PL UK; 5 National Institute for Health Research (NIHR), University College London Hospitals (UCLH), Biomedical Research Centre (BMC), University College London Hospitals, 1st Floor Central, 250 Euston Road, London, NW1 2PG UK; 6 Nuffield Department of Orthopaedics, Rheumatology and Musculoskeletal Sciences, University of Oxford, Oxford, OX3 7LD UK; 7 Nuffield Department of Surgical Sciences, University of Oxford, Oxford, OX3 9DU UK; 8 National Institute for Health Research Collaboration for Leadership in Applied Health Research and Care (NIHR CLAHRC) West, University Hospitals Bristol NHS Trust, 9th Floor, Whitefriars, Lewins Mead, Bristol, BS1 2NT UK

**Keywords:** Randomised controlled trials, Informed consent, Recruitment, Comprehension, Psychometrics

## Abstract

**Background:**

Informed consent (IC) is an ethical and legal prerequisite for trial participation, yet current approaches evaluating participant understanding for IC during recruitment lack consistency. No validated measure has been identified that evaluates participant understanding for IC based on their contributions during consent interactions. This paper outlines the development and formative evaluation of the Participatory and Informed Consent (PIC) measure for application to recorded recruitment appointments. The PIC allows the evaluation of recruiter information provision and evidence of participant understanding.

**Methods:**

Published guidelines for IC were reviewed to identify potential items for inclusion. Seventeen purposively sampled trial recruitment appointments from three diverse trials were reviewed to identify the presence of items relevant to IC. A developmental version of the measure (DevPICv1) was drafted and applied to six further recruitment appointments from three further diverse trials to evaluate feasibility, validity, stability and inter-rater reliability. Findings guided revision of the measure (DevPICv2) which was applied to six further recruitment appointments as above.

**Results:**

DevPICv1 assessed recruiter information provision (detail and clarity assessed separately) and participant talk (detail and understanding assessed separately) over 20 parameters (or 23 parameters for three-arm trials). Initial application of the measure to six diverse recruitment appointments demonstrated promising stability and inter-rater reliability but a need to simplify the measure to shorten time for completion. The revised measure (DevPICv2) combined assessment of detail and clarity of recruiter information and detail and evidence of participant understanding into two single scales for application to 22 parameters or 25 parameters for three-arm trials. Application of DevPICv2 to six further diverse recruitment appointments showed considerable improvements in feasibility (e.g. time to complete) with good levels of stability (i.e. test-retest reliability) and inter-rater reliability maintained.

**Conclusions:**

The DevPICv2 provides a measure for application to trial recruitment appointments to evaluate quality of recruiter information provision and evidence of patient understanding and participation during IC discussions. Initial evaluation shows promising feasibility, validity, reliability and ability to discriminate across a range of recruiter practice and evidence of participant understanding. More validation work is needed in new clinical trials to evaluate and refine the measure further.

**Electronic supplementary material:**

The online version of this article (doi:10.1186/s13063-017-2048-7) contains supplementary material, which is available to authorized users.

## Background

Informed consent (IC) is a legally and ethically established prerequisite for trial participation that is enshrined in international and national guidelines [1–3]. IC is defined as having five elements, all of which are required for consent to be regarded as legal and ethical: capacity, disclosure, understanding, voluntariness and permission [4]. Whilst the content and quality of written patient information is closely monitored by institutional review boards and ethics committees [5–7], less attention is paid to evaluating the quality of information provided in recruitment appointments [8]. Yet, face-to-face discussion is of pivotal importance in informed consent: systematic reviews (SRs) have demonstrated its value for optimising understanding during the consent process with the strongest evidence for improved understanding associated with extended discussion or enhanced Consent Forms [9, 10]. The most recent SR [9] highlighted the heterogeneity of studies reviewed and called for the standardisation of approaches to evaluating IC and consistency in assessing participant understanding for IC.

Existing methods for assessing participant understanding for IC for research participation include participant self-report via questionnaires [11–13] or structured telephone interviews [14, 15] and evaluations of the recruitment discussion [8]. Most rely on participant recall, but vary in what they attempt to measure, including actual understanding [11, 13–15], perceived understanding [11, 12] and satisfaction with the IC process [12, 14]. Frameworks to encourage systematic implementation of best practice by trial recruiters during consent interactions have also been proposed [8, 16–20]. These set out to measure [8, 17] or evaluate [19, 20] recruiter behaviour and propose models of best practice during the interaction [8, 16, 17, 19], the ultimate goal being to improve participant understanding and protect against coercion.

These frameworks evaluate the content and manner in which recruiters provide information, but make no attempt to measure participant understanding as demonstrated in the interaction or extent of participation in the conversation [4, 21]. Evidence over three decades shows that both consent to, and refusal of, trial participation continues to occur despite suboptimal understanding by patients of what participation entails [21–23]. Evidence continues to emerge that trial recruitment is challenging [24] and essential trial concepts, such as randomisation and equipoise, are often not fully understood by trial participants [25–32]. Recruiters need to be able to judge whether a participant has understood the information provided [2] and this judgement by the recruiter will usually be formed on the basis of patient contributions during consent discussions. Evidence of participant understanding (or misunderstanding) as it emerges during recruitment appointments is, therefore, fundamental in evaluating the quality of information provision by the recruiter. A number of studies have shown that recruiters need support to optimise their approaches to information provision during recruitment [18–20, 25, 33–37]. A measure which aims to evaluate what and how information is provided by recruiters and, at the same time, what evidence there is of understanding by patients, will not only provide evidence of patient understanding for the first time, but also allow insight into recruiter behaviour that may then be amenable to feedback and/or training to ensure that recruiters attend to patient understanding during consent interactions.

We set out to develop a measure of IC that could be applied to consent interactions taking place during recruitment appointments (or recordings of these) to evaluate the breadth and clarity of information provision on key issues required for IC and also, more innovatively, to assess evidence of patient understanding and participation during the interaction. The ultimate aim of this work is to optimise participant understanding during trial recruitment by improving recruiter practice during informed consent discussions. This paper outlines the development and formative evaluation of the Participatory and Informed Consent (PIC) measure.

## Methods

There were two stages in the development and formative evaluation of the PIC measure: first, determining items for inclusion; and second a two-phase formative evaluation.

### Determining concepts for inclusion in the PIC measure

The following academic literature was reviewed to identify potential concepts for inclusion: published international and national guidelines on what information should be understood by potential participants for IC to be achieved [1–3, 5–7]; existing measures of understanding for IC [8, 11–15]; and evaluative frameworks to guide recruiters on what and how information should be conveyed during trial recruitment appointments to promote shared decision-making or patient-centred discussion about trial participation [16–20]. From these reviews, an initial list of concepts and potential items was derived (Table 1).

**Table 1:** Core concepts identified for inclusion in the recruitment appointments

• Consultation purpose	
• Relevant history, diagnosis and/or management to date	
• Current management options (independent of study)	
• Clinical Equipoise regarding trial treatments	
• Research study purpose or question	
• Trial arm 1 processes, disadvantages/risks, advantages/benefits	
• Trial arm 2 processes, disadvantages/risks, advantages/benefits	
• (3 arm trial only) Trial arm 3 processes, disadvantages/risks, advantages/benefits	
• Reason for trial or trial purpose	
• Randomisation	
◦ *Reason for randomisation*	
◦ *Process of randomisation*	
• Detail on trial treatment options	
◦ *Processes, potential risks and benefit*	
• Detail on trial procedures	
◦ *Potential risks/costs/burden & benefits of taking part,*	
◦ *Options to refuse or withdraw,*	
◦ *Options for further support in decision making about participation,*	
◦ *Benefits to professional or organisation of P taking part.*	
◦ *Confidentiality of data*	
◦ *Explanation re compensation arrangements*	

Revised framework of core concepts following application to 17 recruitment consultations from 3 diverse trials

A purposive sample of 17 audio-recorded recruitment appointments involving recruiters with a range of specialties and backgrounds (seven surgeons, two oncologists and eight nurses) from three trials [38–41] (cancer/noncancer, two- and three-arm trials, including surgical/nonsurgical arms) was selected to obtain a wide range of appointment types including the outcomes of accepted or refused random allocation of treatment or being undecided and requesting further time or consultation to support decision-making about trial participation (Table 2).

**Table 2:** Characteristics of trials and sample recruitment appointments

Trial	Development of DevPICv1 *N* = 17			Phase 1 evaluation *N* = 6 (DevPICv1)						Phase 2 evaluation *N* = 6 (DevPICv2)					
	CLASS	CHEMORAD	ProtecT	POUT		CSAW		OPTIMA		ACST II		OPTIMA		ProtecT	
*N* appointments	6	3	8	3		2		1		2		2		2	
Blinded category Appt ID:1–6				Less good 3.	Good 1. 2.	Less good 4.	Good 5.	Less good 6.	Good -	Less good 2.	Good 1.	Less good 4.	Good 3.	Less good 5.	Good 6.
Cancer (Y/N)	N	Y	Y	Y		N		Y		N		Y		Y	
Surgical arm (Y/N)	Y	Y	Y	N		Y		N		Y		N		Y	
*N* of trial arms	2	2	3	2		3		2		2		2		3	
Recruiter															
Surgeon	5	1	1	N/A		2		N/A		2		N/A		0	
Oncologist	N/A	2	0	3		N/A		1		N/A		2		0	
Nurse	1	0	7	0		0		0		0		0		2	
Outcome for trial participation Yes, No or Undecided^a^	N = 0	N = 0	N = 5	No = 2		No = 1		N = 1		N = 0		No = 0		N = 1	
	Y = 0	Y = 0	Y = 2	Y = 1		Y=0		Y=0		Yes = 1		Y = 0		Y = 1	
	Undecided = 6	Undecided = 3	Undecided = 1	Undecided =0		Undecided =1		Undecided =0		Undecided = 1		Undecided =2		Undecided =0	
Length of consultation	Mean:13 m 49 s Range: 8 m 58 s–16 m 22 s	Mean:30 m 46 s Range: 25 m 30s–37 m 55 s	Mean:58 m 24 s Range: 14 m 43 s–1 h 48 m 24 s	39 m 58 s 26 m 43 s 33 m 19 s		16 m 50s 24 m 01 s		31 m 10s		20 m 22 s 14 m 26 s		1 h 06 m 27 s 22 m 13 s		18 m 44 s 43 m 02 s	
Length of time to rate (min)	N/A			163 m 169 m 120 m		75 m 94 m		81 m		73 m 55		51 m 54 m		39 m 65 m	
Mean time to rate (min)	N/A			117 m						56 m					

^a^Undecided = outcome agreed was further time to consider participation and/or another appointment to discuss participation further. All participants were providing consent for themselves

Two researchers with expertise in recruitment to trials (JW and SP) analysed all 17 appointments independently to identify the presence or absence of concepts identified in Table 1 and to identify other concepts that seemed relevant to the IC or interactional process. Findings were compared and discrepancies discussed between JW and SP to reach agreement.

Combining evidence from the academic literature review and subsequent assessment of the practicability and feasibility of evaluating audio-recordings of trial recruitment appointments, a developmental version of the measure was drawn up (DevPICv1, Additional file 1: Appendix A). The measure was designed to be completed whilst listening to the audio-recording of the appointment with a transcript present if required.

### Formative evaluation of the developmental PIC

Formative evaluation of the DevPIC was carried out iteratively in two phases.

#### Phase 1

The DevPICv1 (Additional file 1: Appendix A) was applied to six further recruitment appointments from three different trials [42–44] (Table 2). Appointments were purposively sampled to include surgeon and oncologist recruiters, two- and three-arm trials, with outcomes of trial participation and refusal and where information provision was judged to be ‘good’ or ‘less good’ by researchers involved in qualitative research into trial recruitment (Table 2). The DevPICv1 was applied to appointments by two researchers (JW and DE) blind to these categorisations. Both raters had expertise in recruitment to trials. Raters were asked independently to evaluate the feasibility, validity and reliability of the measure in the following ways:

- *Feasibility*: the time taken to apply the measure to each recruitment appointment was recorded to determine whether it was feasible to continue with it in its existing format. It was assumed that a developmental version should be completed in under an hour.
- *Validity*: initial feedback on the face validity of the measure was obtained through free-text comments and feedback from the raters who completed it. Response rates and missing data were identified for individual items to evaluate their acceptability.
- *Reliability*: inter-rater reliability was assessed by evaluating differences in item responses made independently by the two raters. Each rater was required to rate each of 20 parameters (two-arm trials) or 23 parameters (three-arm trials – see Additional file 1: Appendix A) four times (evaluating first the quantity then the clarity of recruiter information, the quantity of patient talk and then evidence of understanding shown in patient talk about each parameter) giving a total of 80 ratings per appointment (two-arm trials) or 92 ratings per appointment (three-arm trials) respectively.
- *Stability*: the stability (test-retest reliability) of the new measure was assessed by evaluating changes in item responses when the measure was applied to a single appointment by the same researcher, with an interval of at least 14 days. Rating procedure was as described for inter-rater reliability.
- In interpreting both reliability and stability, a discrepancy of 1 point or less was deemed acceptable on the grounds that this might represent the difference between the presence and absence of information and between ‘mostly clear’ and ‘very clear’ on the scale. Larger discrepancies were noted.
- *Qualitative evaluation*: free-text comments recorded by raters on the content and interaction in the recruitment appointment and the application of the measure were collated. Thematic analysis [45] was used to identify emergent themes in relation to the content of the information and patterns of interaction between recruiter and patient (e.g. what and how much each contributed) during discussion.
- Findings from phase 1 were reviewed by a panel (JW, DE, KNLA, JLD, RB) convened to revise and shorten the measure to produce DevPICv2. In phase 2, it was then applied by the same two researchers (JW and DE) to six new appointments, purposively sampled as before from three trials [38, 39, 43, 46], including two appointments led by nurse recruiters, and again including one appointment from each trial where information provision was comprehensive and clear and another where information provision was less comprehensive and clear (Table 2). As before, raters were blind to these categorisations.

#### Phase 2

Ratings were again compared to evaluate feasibility, validity, response rates and missing data, inter-rater reliability and stability (test-retest reliability) as described in phase 1 above.

##### Reliability and Stability

Each rater was required to rate each of 22 parameters (two-arm trials, four appointments) or 25 parameters (three-arm trials, two appointments) twice, first evaluating the presence and clarity of recruiter information and second evidence of understanding shown in patient talk about each parameter. This gave a total of 138 comparisons evaluating recruiter information and 138 comparisons evaluating evidence of patient understanding respectively. As in phase one, a discrepancy of 1 point or less was deemed acceptable on the grounds that this might represent the difference between the presence and absence of information and between ‘mostly clear’ and ‘very clear’ on the scale. Larger discrepancies were noted.

Free-text or narrative comments made during application of the measure were collated and analysed thematically as described above. In addition, concurrent validity was assessed by applying another measure of recruiter information provision for informed consent, the Process and Quality of Informed Consent Instrument (P-QIC) [8], to the same recruitment appointments. The P-QIC evaluates recruiter information provision (rather than both recruiter information provision and evidence of patient understanding) so domains measured in the P-QIC were expected to map most closely onto the domains measured in the recruiter information provision section of the DevPICv2. The Spearman’s rank correlation was, therefore, calculated between the total score on the P-QIC and the total DevPICv2 score for recruiter information provision.

## Results

### Determining items for inclusion in the DevPIC

Analysis of the initial 17 recruitment appointments revealed wide variation in both whether and how concepts identified within guidelines as a pre-requisite for the IC process were presented and discussed during appointments. Guidelines identified concepts to be covered but did not provide sufficient detail to enable consistency in presentation during recruitment to trials in practice. The broad stipulation that participants should understand trial procedures [2, 3] was too general in that some recruiters omitted basic concepts fundamental to randomised controlled trial (RCT) participation, such as the rationale for randomization, and there was evidence that participants were confused by this. It also became apparent that concepts identified in ethical frameworks [2] foregrounded ethical priorities in protecting participant rights and autonomy, but did not necessarily match the specific information needs of individual participants. It was noted that where participants contributed substantively to the discussion, there was evidence that they created meaning and understanding dynamically by combining previous knowledge with new information provided during discussion.

Concepts and items in the measure were revised to reflect these issues and particularly to include patient priorities during the information exchange as identified in these appointments and previous studies [18–20, 25, 47]. The first developmental version of the PIC (DevPICv1) was, therefore, developed to take into account recruiter and patient perspectives of the information required for informed consent.

### Formative evaluation of the DevPIC

#### Phase 1

Table 2 shows the characteristics of the six recruitment appointments to which the measure was applied by two researchers (JW and DE) during the first phase of evaluation [42–44].

##### Feasibility of the measure

Completion of the measure took a mean of 117 min per appointment, and varied from 75 to 169 min for those lasting between 17 min and 40 min. Although raters felt that the time commitment decreased as familiarity with the measure increased this was longer than the hour limit set; therefore, the measure needed to be reduced.

##### Validity of the measure

Analysis of missing data for individual items showed no missing data in first application of the measure by either rater, indicating good acceptability of included items. Free-text comments provided by raters reported difficulty rating levels of patient understanding.

##### Reliability of the measure

Levels of inter-rater agreement are shown in Table 3. Across the appointments, inter-rater agreement showed a discrepancy of 1 point or less ranging between 113/126 (89.68%: patient talk) and 89/126 (70.63%: patient understanding). Higher levels of inter-rater agreement were observed for ratings of quantity of recruiter information provision than for ratings of clarity of recruiter information and for ratings of how much a patient talked about a topic than for ratings of their understanding (Table 3).

**Table 3:** Phase 1 evaluation of inter-rater reliability and test-retest stability

	Recruiter info–quantity	Recruiter info– clarity	Patient talk–quantity	Patient talk–understanding
Phase 1: inter-rater reliability				
*N* of comparisons	126	126	126	126
*N* of comparisons showing ≤1-point discrepancy (% of total comparisons)	111 (88.10)	92 (73.02)	113 (89.68)	89 (70.63)
*N* of comparisons showing 2-point discrepancy (% of total comparisons)	13 (10.32)	5 (3.97)	10 (7.94)	7 (5.56)
*N* of comparisons showing ≥3-point discrepancy (% of total comparisons)	2 (1.59)	29 (23.02)^a^	3 (2.38)	30 (23.81)^a^
^a^Includes those comparisons where N/A (coded 9) was marked for one application of the measure and not the other				
Phase 1: test-retest stability				
*N* of comparisons	126	126	126	126
*N* of comparisons showing ≤1-point discrepancy (% of total comparisons)	124 (98.41)	116 (92.06)	124 (98.41)	114 (90.48)
*N* of comparisons showing 2-point discrepancy (% of total comparisons)	2 (1.59)	1 (0.79)	2 (1.59)	1 (0.79)
*N* of comparisons showing ≥3-point discrepancy (% of total comparisons)^a^	0 (0.00)	9 (7.14)^a^	0 (0.00)	11 (8.73)^a^

^a^Includes those comparisons where N/A (coded 9) was marked by one rater and not the other

##### Stability of the measure

Rates of test-retest or intra-rater agreement are shown in Table 3. Across four appointments for two-arm trials and two appointments for three-arm trials, test-retest agreement showed a discrepancy of 1 point or less ranging between 124/126 (98.41%: quantity of recruiter information provision and patient talk) and 114/126 (90.48%: patient understanding, Table 3). As with inter-rater agreement, higher levels of test-retest agreement were observed for ratings of quantity of recruiter information provision than for ratings of clarity and for ratings of how much a patient talked about a topic than for ratings of their understanding (Table 3).

##### Qualitative evaluation

Free-text comments noted on application of the measure during phase 1 of the evaluation highlighted a number of issues. Time taken to complete the measure needed to be reduced and it was questioned whether a rater was able to judge levels of patient understanding *per se* on the basis of evidence emerging from the interaction. It was argued that a more realistic option would be to rate only *evidence* of understanding or misunderstanding. Recruiters who were most successful in allowing evidence of understanding to emerge during the discussion facilitated substantive patient contributions to the discussion. They also framed equipoise as providing the rationale for both the trial and the random allocation of treatment to take place.

Review of these findings by the panel resulted in the revised version presented in Additional file 1: Appendix B. Changes made to the measure were as follows:

1. Levels of recruiter detail and clarity of detail were combined into a single scale so that raters were able to rate presence/absence of information and the level of clarity of that information within a single 4-point scale (0 = absent, 1 = mostly unclear, 2 = mostly clear, 3 = very clear).
2. The two scales rating the level of detail found in patient talk and levels of patient understanding were also merged into a single 4-point scale. Feedback from the qualitative evaluation was that raters could not be expected to make a judgement on participants’ levels of understanding but only to judge levels of *evidence* of understanding. The revised scale required a judgement about levels of evidence of understanding (0 = evidence of misunderstanding which was left unclarified by the end of the appointment, 1 = no evidence of understanding, 2 = minimal evidence of understanding, e.g. agreement tokens such as ‘ok’, ‘mm’, ‘I see’, 3 = substantive evidence of understanding which might be provided by a patient comment, e.g. '*well he said it's possible that it [RCT intervention] could cause a stroke*’).
3. Key content relevant for setting the trial in the context of the patient’s diagnosis and decision-making regarding treatments were brought together in Section 2i, items 1–8 of the measure (Additional file 1: Appendix B).
4. Items relating to processes of randomisation (items 16, 17 and 18 Additional file 1: Appendix A) were subsumed into a single item evaluating process of randomisation (item 8, Additional file 1: Appendix B).
5. Four items were incorporated (Additional file 1: Appendix B): item 4 assessed the presentation of management options within the trial separately from the management options available generally; item 23 assessed description of any benefits to the professional should the patient choose participation; item 24 assessed the description of measures to protect patient confidentiality; and item 25 assessed description of measures for patient compensation in case of adverse events. All these items had been previously implicitly assessed within other parameters but were judged to need explicit assessment in their own right.
6. A section containing four global judgements was added to the measure (Additional file 1: Appendix B, Section 3). Raters were required to judge whether the recruiter consistently conveyed equipoise; whether the patient was in equipoise by the end of the appointment (or when any decision about participation took place); whether the patient accepted random allocation as a means to determine treatment; and whether the patient appeared sufficiently informed by the end of the appointment (or when any decision about participation took place) to make an informed decision on participation. For each of these the judgement was a binary choice between ‘yes’ or ‘no’ with the option to record that there was insufficient evidence to make a judgement and space for free-text comments.
7. Raters were invited to provide free-text comments on the following aspects of the appointment: (1) what the recruiter said, (2) how it was said and (3) how it appeared to be understood by the participant (Additional file 1: Appendix B, Section 4).

It was expected that these changes would result in a substantial reduction in time taken to administer the measure without reducing validity and inter-rater reliability.

#### Phase 2

The DevPICv2 (Additional file 1: Appendix B) was applied to a further six diverse appointments [38, 39, 43, 46] (Table 2) to assess feasibility, validity, stability, inter-rater reliability with a parallel qualitative assessment.

##### Feasibility

Time taken to complete the DevPICv2 is shown in Table 2. The mean completion time (56 min) was less than half that recorded during phase 1.

##### Validity

Analysis of missing data for individual items showed no missing data in the first application of the measure by either rater, indicating good acceptability of included items. Spearman’s rank correlation between P-QIC and the total score for DevPICv2 recruiter information provision was 0.80 (*p* = 0.2).

##### Reliability

Inter-rater reliability is shown in Table 4. Inter-rater agreement showed a discrepancy of 1 point or less in 125/138 (90.58%) of ratings of both recruiter information provision and evidence of patient understanding (Table 4).

**Table 4:** Phase 2 evaluation of inter-rater reliability and test-retest stability

	Recruiter information provision	Evidence of patient understanding
Phase 2: inter-rater reliability		
*N* of comparisons	138	138
*N* of comparisons showing ≤1-point discrepancy (% of total comparisons)	125 (90.58)	125 (90.58)
*N* of comparisons showing 2-point discrepancy (% of total comparisons)	10 (7.25)	13 (9.42)
*N* of comparisons showing ≥3-point discrepancy (% of total comparisons)	3 (2.17)	0 (0.00)
Phase 2: test-retest stability		
*N* of comparisons	138	138
*N* of comparisons showing ≤1-point discrepancy (% of total comparisons)	137 (99.28)	137 (99.28)
*N* of comparisons showing 2-point discrepancy (% of total comparisons)	0 (0.00)	1 (0.72)
*N* of comparisons showing ≥3-point discrepancy (% of total comparisons)	1 (0.72)	0 (0.00)

##### Stability of the measure

Rates of test-retest or intra-rater agreement are shown in Table 4. Test-retest agreement showed a discrepancy of 1 point or less in 137/138 (99.28%) of ratings of both recruiter information provision) and evidence of patient understanding (Table 4).

##### Analysis of global judgements

**Fig. 1:**
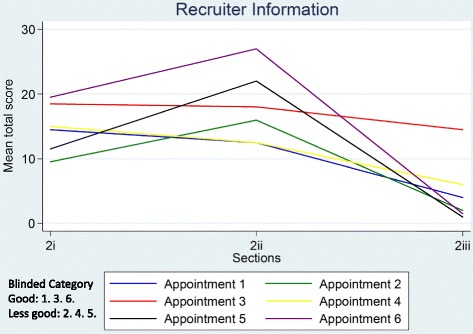
Mean total section scores for recruiter information provision

**Fig. 2:**
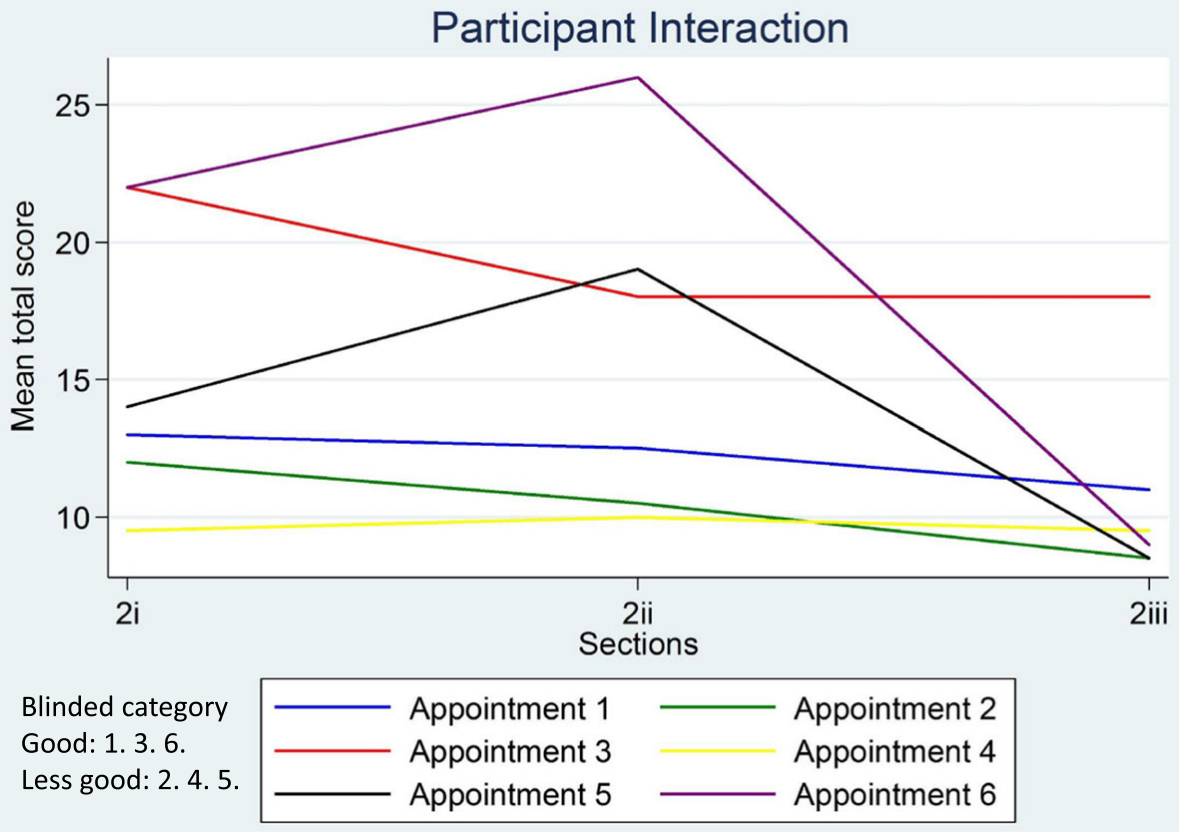
Mean total section scores for participant interaction

Ratings of global judgements (Section 3) are shown in Table 5. Appointments 3 and 6 were judged by both raters to show sufficient understanding for informed consent; remaining appointments were judged to show insufficient evidence for informed consent by both (2, 4 and 5) or one (1) rater. When ratings on the global judgement ‘*evidence of sufficient understanding for informed consent*’ were compared to the DevPICv2 total scores for Sections 2i-2iii across each appointment (Figs. 1 and 2) there was broad agreement between ratings on the former and the latter. DevPICv2 scores show appointments 3 and 6 scoring highly and appointments 1, 2, 4 and 5 scoring more poorly. A higher DevPICv2 score was recorded for appointment 5 on Section 2ii (describing treatment processes, risks and benefits) but scores for this appointment on Sections 2i and 2iii were comparable to appointments 1, 2 and 4.

**Table 5:** Global judgements from DevPICv2 Section 3

Appointment	Rater	Does the recruiter consistently convey a position of equipoise and what evidence do you have to suggest this?	Is the P in equipoise^a^ and what evidence do you have to suggest this?	Does the patient accept randomisation as a means to determine treatment^a^ and what evidence do you have to support this?	Is there evidence that the patient is sufficiently informed by the end of the consultation to make an informed decision and what evidence do you have to support this?
1	1	Y	Unclear	Unclear	Unclear
	2	Y	Y	Y	Y
2	1	N	Unclear	Unclear	N
	2	N	Y	Unclear	N
3	1	Y	Y	Unclear	Y
	2	Y	Y	Y	Y
4	1	N	Unclear	N	N
	2	N	Unclear	N	N
5	1	Y	Unclear	N	Unclear
	2	N	Unclear	N	Unclear
6	1	Y	Y	Y	Y
	2	Y	Y	Y	Y

^a^at the point of decision-making about participation or at the end of the appointment

Y = Yes, N = No, Unclear = insufficient evidence to make this judgement

##### Qualitative evaluation

Comments in this section noted that at times a brief summary of key information (evaluated in Section 2i, DevPICv2) appeared more beneficial to patient understanding than extensive detail about treatment arms (evaluated in Section 2ii). Raters observed that evidence of understanding as created during conversation could be distinguished from evidence of understanding brought by patients and present from the outset. Evidence for this could be detected in patient comments showing awareness of issues that had not yet been discussed or showing evidence of understanding emerging during discussion.

## Discussion

This study describes the development and formative evaluation of a measure of participatory and informed consent (PIC) for application to trial recruitment appointments to evaluate consent interactions for the content and clarity of recruiter information provision and also the extent to which evidence of patient understanding emerges. Initial work identified concepts for inclusion in the measure. This was followed by a two-phase evaluation: phase 1 highlighted a need to shorten the measure to improve feasibility, validity and reliability; and phase 2 showed considerable improvements in feasibility (e.g. time to complete) stability (i.e. test-retest reliability) and inter-rater reliability, suggesting that the measure is now ready for a more comprehensive evaluation. The measure’s novelty and value lie in its evaluation of evidence of patient understanding to place this at the forefront and as the key variable for evaluating the immediate outcomes of an IC discussion. It is now available for further validation in the context of new clinical trials.

The ultimate goal of this work is to optimise information provision by trial recruiters so that they are able to attend to and maximise participant understanding during consent interactions during recruitment. Participant understanding is an ethical prerequisite for recruiters taking consent for trial participation and it should, therefore, be in evidence during the IC discussion prior to consent or refusal being given [2]. By evaluating evidence of understanding in the context where understanding is in part created, the measure draws attention to the imperative for the recruiter to have such evidence available, avoids problems with recall and allows us to identify approaches to recruiter information provision that facilitate or inhibit understanding. Such insights will be used to guide recruiter training. Initial evaluation shows promising feasibility, validity and reliability and evidence that the measure is able to discriminate across a range of recruiter practice and evidence of participant understanding.

Previous measures of informed consent for research have mainly used methods of participant reporting via questionnaires or telephone interviews [11–15]. Such measures enable identification of areas of understanding (or lack of it) but do not offer insight on how or why failures of understanding arise [10]. Failures of understanding may be the result of an issue not having been discussed, not having been discussed clearly, having been discussed but not properly understood, or recall problems. One existing measure, the P-QIC [8], has attempted to circumvent such issues by quantifying the IC process as it occurs during consent discussions. The P-QIC shares the advantages of the PIC of evaluating both content and manner of information provision by the recruiter and capturing this as it takes place, rather than as it is recalled by the participant [8]. Relatively high correlations were found between P-QIC and DevPICv2 scores for recruiter information provision, implying that the P-QIC and the DevPICv2 evaluate common domains in terms of content and manner of recruiter information provision. However, correlations did not reach significance, possibly due to the small sample size. Furthermore the P-QIC does not evaluate evidence of participant understanding in the interaction [8].

This study describes the development and formative evaluation of the PIC; further evaluation is ongoing. Mean time to complete the measure remained at just under an hour per appointment during phase-2 evaluation: for the measure to be feasible for application in busy clinics it will benefit from further reduction in rater burden. Evaluation to date has been small scale and data on stability and reliability have been reported as percentage agreements rather than performing formal calculations (e.g. kappa statistics). Future evaluation should include sociodemographic data on patients involved in the recruitment appointments. However, the PIC evaluates understanding for IC in a way that has not previously been attempted and an iterative developmental process was most appropriate at this stage. There are methodological challenges in attempting to measure evidence of participant understanding (or misunderstanding) based on their contributions to a discussion, e.g. assuming understanding on the basis of a minimal response (e.g. ‘mm’ or ‘uhuh’) from the participant to information presented by the recruiter when they may have been functioning as continuers, signalling ‘passive recipiency’ [48].

Insights from conversation analysis (CA) on achieving understanding in interaction show that repeat utterances or even statements such as ‘I understand’ are often treated as only claiming, as opposed to evidencing understanding [49]. Further evidence may be needed to confirm levels of patient understanding in addition to the current DevPICv2 approach to evaluating these minimal responses. Reaching optimum understanding for IC is best conceptualized as a process rather than a single event [50] extending beyond a single recruitment appointment and involving discussions with different health professionals and several sources of information including formal and informal written information and discussions with family and friends [51]. However, recruitment appointments remain the focal point where recruiters have an ethical imperative to explore and address gaps in understanding and gain written informed consent to participate. Although the measure has been designed to be applied to a single IC appointment, where multiple appointments occur for a single patient, it could be applied repeatedly to capture differences between appointments. Inevitably the measure’s value depends on the rater having access to an audio- or video-recording of the entire appointment and recordings may be started late or recorders switched off with important discussion unrecorded. Our study employed audio-recording which fails to capture nonverbal communication which has not been included in our analysis.

Future development of the measure will include the creation of a detailed coding manual with the aim of increasing inter-rater reliability and evaluation of the measure’s performance in additional trial contexts in a full psychometric evaluation. Promising agreement was shown in DevPICv2 between evaluation using Section 2i-iii and Section 3 and it may be that the measure can be reduced further using Section 3 as a model; more data are needed to determine relative validity of these two sections. The measure currently incorporates a qualitative narrative describing the interaction, adding to the rater burden but providing useful insights into issues that are highly relevant to optimizing patient understanding. Future work will examine whether these areas can be quantified. Preliminary evaluation reported here showed variation in the extent to which recruiter information provision conformed to standards identified in our measure and assumed to be requisite for the IC process, indicating promising discriminative validity and showing consistency with other study findings [8]. It also showed variation in the extent to which evidence of participant understanding emerged. We cannot claim that poor conformity with these standards of recruiter information provision necessarily indicates a failed IC process. Further research is needed to establish the relationship between recruiter information provision and evidence of patient understanding as measured by the PIC, but also as measured by self-report measures of IC. Nor is it clear whether individual items identified in the PIC have disproportionately large impact on patient understanding for trial participation; it cannot be assumed that all items will have equal impact on understanding for all individuals.

The PIC was conceived as a formative measure of informed consent, i.e. application of the measure will inform on areas of recruiter practice that can be modified in order to benefit participant understanding for informed consent. The measure provides a rapid method of evaluating the breadth and clarity of information provided by recruiters in order to highlight areas where information is lacking or unclear with a view to feed this information into training of recruiters. It was designed to be applicable across trials and diseases so as to be maximally generalizable [52]. Our approach marks a move away from a disclosure model of IC towards a participatory model of IC which shares some of the premises of shared decision-making [53]. It is important that recruiters understand that engaging patients in discussion about their preference is not coercive but can be an essential prerequisite for the informed consent process [47, 54].

## Conclusion

The DevPICv2 provides a novel measure of IC that can be applied directly to recruitment appointments where trial participation is discussed in order to evaluate the quality of recruiter information provision and, most importantly for consent interactions, evidence of patient understanding. Initial evaluation shows promising feasibility, validity and reliability and evidence that the measure is able to discriminate across a range of recruiter practice and evidence of participant understanding. Further validation work is needed in new clinical trials to evaluate and refine the measure further.

## Additional file

Additional file 1:
**Appendix A.** DevPICv1. Participatory and Informed Consent for trial recruitment. **Appendix B.** DevPICv2. Participatory and Informed Consent for trial recruitment. (PDF 533 kb)

## Abbreviations

DevPICv1: Developmental version of the measure of Participatory and Informed Consent version 1
DevPICv2: Developmental version of the measure of Participatory and Informed Consent version 2
IC: Informed consent
NHS: National Health Service
PIC: Participatory and Informed Consent
P-QIC: Process and Quality of Informed Consent Instrument
RCT: Randomised controlled trial
SR: Systematic review
UK: United Kingdom
