# Supplementary material for: Informed consent in randomised controlled trials: development and preliminary evaluation of a measure of Participatory and Informed Consent (PIC)
Source: Trials. 2017 Jul 17;18:327. doi: 10.1186/s13063-017-2048-7 (PMC5513045; doi:10.1186/s13063-017-2048-7)
Supplement: Additional file 1: — Appendix A. DevPICv1. Participatory and Informed Consent for trial recruitment. Appendix B. DevPICv2. Participatory and Informed Consent for trial recruitment. (PDF 533 kb) [file 13063_2017_2048_MOESM1_ESM.pdf]

Additional file 1

Appendix A **DevPICv1**

Participatory and Informed Consent for trial recruitment

Section A: Descriptive information about the consultation

Consultation reference		Rater's initials	
Recruiter ID		Date of rating	
Time taken to rate		Length of consultation if known	
Trial treatment arms	1.<br>2.<br>3.	Decision outcome in terms of participation (if known):	
Comments e.g. analysis process, nature of consultation, quality of audio recording			

IC = informed consent **P= Patient**

R= recruiter **P2=significant other accompanying patient**

R2 = second recruiter

Section B Detailed analysis of R information provision and P understanding

	Start time (h:m)	Finish time (h:m)	Total time (h:m)
**Total time taken to rate all of section C (parts i-iii)**			

i) Diagnosis & management options

Topic	R covers information on:<br>Not at all Very much so	Clarity of R information on:<br>Not at all clear Very clear	P talks about:<br>Not at all Very much so	Based on evidence of Ps contributions, P understands information on:<br>Not at all Very much so
**1. Purpose of consultation**	**1a**<br>**0 1 2 3**<br>***If 0 mark next column N/A***	**1b**<br>**0 1 2 3**<br>**N/A**	**1c**<br>**0 1 2 3**<br>***If 0 mark next column N/A***	**1d**<br>**0 1 2 3**<br>**N/A**
**2. Relevant history: diagnosis and management to date**	**2a**<br>**0 1 2 3**<br>***If 0 mark next column N/A***	**2b**<br>**0 1 2 3**<br>**N/A**	**2c**<br>**0 1 2 3**<br>***If 0 mark next column N/A***	**2d**<br>**0 1 2 3**<br>**N/A**
**3. Current management options**<br>**(independent of study)**	**3a**<br>**0 1 2 3**<br>***If 0 mark next column N/A***	**3b**<br>**0 1 2 3**<br>**N/A**	**3c**<br>**0 1 2 3**<br>***If 0 mark next column N/A***	**3d**<br>**0 1 2 3**<br>**N/A**
**4.Clinical equipoise: no evidence to suggest any treatment will give a better outcome than other(s)**	**4a**<br>**0 1 2 3**<br>***If 0 mark next column N/A***	**4b**<br>**0 1 2 3**<br>**N/A**	**4c**<br>**0 1 2 3**<br>***If 0 mark next column N/A***	**4d**<br>**0 1 2 3**<br>**N/A**
**Total score n /12**	**/12**	**/12**	**/12**	**/12**
**Comments**				

ii) Study purpose & treatments

Topic	R covers information on:<br><br>Not at all Very much so	Clarity of R information on:<br><br>Not at all clear Very clear	P talks about:<br><br>Not at all Very much so	Based on evidence of Ps contributions, P understands information on:<br>Not at all Very much so
5. Research study purpose or question	5a<br>0 1 2 3<br>*If 0 mark next column N/A*	5b<br>0 1 2 3<br>N/A	5c<br>0 1 2 3<br>*If 0 mark next column N/A*	5d<br>0 1 2 3<br>N/A
6.Trial arm 1 processes<br>……………………<br>(*Name treatment*)	6a<br>0 1 2 3<br>*If 0 mark next column N/A*	6b<br>0 1 2 3<br>N/A	6c<br>0 1 2 3<br>*If 0 mark next column N/A*	6d<br>0 1 2 3<br>N/A
7.Trial arm 1 disadvantages or risks	7a<br>0 1 2 3<br>*If 0 mark next column N/A*	7b<br>0 1 2 3<br>N/A	7c<br>0 1 2 3<br>*If 0 mark next column N/A*	7d<br>0 1 2 3<br>N/A
8.Trial arm 1 advantages or benefits	8a<br>0 1 2 3<br>*If 0 mark next column N/A*	8b<br>0 1 2 3<br>N/A	8c<br>0 1 2 3<br>*If 0 mark next column N/A*	8d<br>0 1 2 3<br>N/A
9.Trial arm 2 processes<br>……………………<br>(*Name treatment)*	9a<br>0 1 2 3<br>*If 0 mark next column N/A*	9b<br>0 1 2 3<br>N/A	9c<br>0 1 2 3<br>*If 0 mark next column N/A*	9d<br>0 1 2 3<br>N/A
10.Trial arm 2 disadvantages or risks	10a<br>0 1 2 3<br>*If 0 mark next column N/A*	10b<br>0 1 2 3<br>N/A	10c<br>0 1 2 3<br>*If 0 mark next column N/A*	10d<br>0 1 2 3<br>N/A
11.Trial arm 2 advantages or benefits	11a<br>0 1 2 3<br>*If 0 mark next column N/A*	11b<br>0 1 2 3<br>N/A	11c<br>0 1 2 3<br>*If 0 mark next column N/A*	11d<br>0 1 2 3<br>N/A
12.Trial arm 3 processes<br>N/A<br>Name…………………<br>If N/A go to Q15	12a<br>0 1 2 3<br>*If 0 mark next column N/A*	12b<br>0 1 2 3<br>N/A	12c<br>0 1 2 3<br>*If 0 mark next column N/A*	12d<br>0 1 2 3<br>N/A
13.Trial arm 3 disadvantages or risks	13a<br>0 1 2 3<br>*If 0 mark next column N/A*	13b<br>0 1 2 3<br>N/A	13c<br>0 1 2 3<br>*If 0 mark next column N/A*	13d<br>0 1 2 3<br>N/A

Topic	R covers information on:<br><br>Not at all Very much so	Clarity of R information on:<br><br>Not at all clear Very clear	P talks about:<br><br>Not at all Very much so	Based on evidence of Ps contributions, P understands information on:<br>Not at all Very much so
**14.Trial arm 3 advantages or benefits**	**14a**<br>**0 1 2 3**<br>***If 0 mark next column N/A***	**14b**<br>**0 1 2 3**<br>**N/A**	**14c**<br>**0 1 2 3**<br>***If 0 mark next column N/A***	**14d**<br>**0 1 2 3**<br>**N/A**
**Total**<br>**If 2 arm trial = /21**<br>**If 3 arm trial= /30**	**If 2 arm trial = /21**<br>**If 3 arm trial= /30**	**If 2 arm trial = /21**<br>**If 3 arm trial= /30**	**If 2 arm trial = /21**<br>**If 3 arm trial= /30**	**If 2 arm trial = /21**<br>**If 3 arm trial= /30**
**Comments**				

iii Study procedures

Topic	R covers information on:<br><br>Not at all Very much so	Clarity of R information on:<br><br>Not at all clear Very clear	P talks about:<br><br>Not at all Very much so	Based on evidence of Ps contributions, P understands information on:<br>Not at all Very much so
15. Reason for randomisation (e.g. prevent selection bias, produce generalizable results)	15a<br>0 1 2 3<br>*If 0 mark next column N/A*	15b<br>0 1 2 3<br>N/A	15c<br>0 1 2 3<br>*If 0 mark next column N/A*	15d<br>0 1 2 3<br>N/A
16. Process of randomisation (only if explicit that it is a chance allocation score =2+)	16a<br>0 1 2 3<br>*If 0 mark next column N/A*	16b<br>0 1 2 3<br>N/A	16c<br>0 1 2 3<br>*If 0 mark next column N/A*	16d<br>0 1 2 3<br>N/A
17. Option to refuse randomisation (if is a pragmatic trial & participation is possible following treatment choice) N/A	17a<br>0 1 2 3<br>*If 0 mark next column N/A*	17b<br>0 1 2 3<br>N/A	17c<br>0 1 2 3<br>*If 0 mark next column N/A*	17d<br>0 1 2 3<br>N/A
18. Implications of randomisation for P (e.g. removes choice but takes away dilemma)	18a<br>0 1 2 3<br>*If 0 mark next column N/A*	18b<br>0 1 2 3<br>N/A	18c<br>0 1 2 3<br>*If 0 mark next column N/A*	18d<br>0 1 2 3<br>N/A
19. Benefits of being in study (e.g. longer follow-up, people in studies fare better)	19a<br>0 1 2 3<br>*If 0 mark next column N/A*	19b<br>0 1 2 3<br>N/A	19c<br>0 1 2 3<br>*If 0 mark next column N/A*	19d<br>0 1 2 3<br>N/A
20. Costs or burden of being in study (e.g. additional appts, samples, questionnaires, duration of commitment)	20a<br>0 1 2 3<br>*If 0 mark next column N/A*	20b<br>0 1 2 3<br>N/A	20c<br>0 1 2 3<br>*If 0 mark next column N/A*	20d<br>0 1 2 3<br>N/A
21. Options for further consultations in decision making re trial participation	21a<br>0 1 2 3<br>*If 0 mark next column N/A*	21b<br>0 1 2 3<br>N/A	21c<br>0 1 2 3<br>*If 0 mark next column N/A*	21d<br>0 1 2 3<br>N/A
22. Option to refuse or withdraw from participation at any time	22a<br>0 1 2 3<br>*If 0 mark next column N/A*	22b<br>0 1 2 3<br>N/A	22c<br>0 1 2 3<br>*If 0 mark next column N/A*	22d<br>0 1 2 3<br>N/A

23. Outlines benefits (financial & other) to professional/organisation of P participation<br><br>N/A	**23a**<br><br>**0 1 2 3**<br><br>***If 0 mark next column N/A***	**23b**<br><br>**0 1 2 3**<br><br>**N/A**	**23c**<br><br>**0 1 2 3**<br><br>***If 0 mark next column N/A***	**23d**<br><br>**0 1 2 3**<br><br>**N/A**
**Totals n/27**	**/27**	**/27**	**/27**	**/27**
**Comments**				

Appendix B **DevPICv2**

Participatory and Informed Consent for trial recruitment

Section 1: Descriptive information about the consultation

Rater ID		Date of rating	
Total time taken to rate	Start time (h:m)	Finish time (h:m)	Total time (h:m)
Consultation ID		Trial ID	
Recruiter ID		Recruiter's Profession	
Length of consultation (h:m)		People present<br>R1 = recruiter<br>R2 = second recruiter<br>P1 = patient<br>P2= friend/family	
Trial treatment arms	1.		
	2.		
	3.		
Decision outcome in terms of participation	e.g. Unknown / Randomised & accepted allocation / Randomised & took time to consider / Randomised & rejected allocation / Refused randomisation & chose treatment / Undecided		

	Scene setting	Study treatments	Study procedures	
Score R info provision	Section 2 i a /24	Section 2 ii a /18 /27	Section 2 iii a /24	Total /60 /69
Score P understanding	Section 2 i a /24	Section 2 ii a /18 /27	Section 2 iii a /24	Total /60 /69

Comments e.g. analysis process, nature of consultation, quality of audio recording

Section 2: R information provision and evidence of P understanding

i) Scene setting: consultation purpose, relevant history/diagnosis, management options, equipoise, reason for trial/randomisation

**Topic**	**a. R information provision is:**				**b. P contributions suggest:** (circle most appropriate box)	
1. Purpose of consultation	0 Absent	1 Mostly unclear	2 Mostly clear	3 Very clear	Evidence of misunderstanding	0
					No evidence available	1
					Minimal evidence of understanding	2
					Adequate evidence of understanding	3
2. Relevant history: diagnosis and management to date	0 Absent	1 Mostly unclear	2 Mostly clear	3 Very clear	Evidence of misunderstanding	0
					No evidence available	1
					Minimal evidence of understanding	2
					Adequate evidence of understanding	3
3. Currently available management options within standard care	0 Absent	1 Mostly unclear	2 Mostly clear	3 Very clear	Evidence of misunderstanding	0
					No evidence available	1
					Minimal evidence of understanding	2
					Adequate evidence of understanding	3
4. Management options evaluated within trial	0 Absent	1 Mostly unclear	2 Mostly clear	3 Very clear	Evidence of misunderstanding	0
					No evidence available	1
					Minimal evidence of understanding	2
					Adequate evidence of understanding	3
5.Clinical equipoise (no evidence to suggest any treatment is better than other(s))	0 Absent	1 Mostly unclear	2 Mostly clear	3 Very clear	Evidence of misunderstanding	0
					No evidence available	1
					Minimal evidence of understanding	2
					Adequate evidence of understanding	3
6. Trial purpose or question (collect evidence as to whether one treatment is better than other(s))	0 Absent	1 Mostly unclear	2 Mostly clear	3 Very clear	Evidence of misunderstanding	0
					No evidence available	1
					Minimal evidence of understanding	2
					Adequate evidence of understanding	3
7. Reason for randomisation (create two groups the same except for which treatment each group gets)	0 Absent	1 Mostly unclear	2 Mostly clear	3 Very clear	Evidence of misunderstanding	0
					No evidence available	1
					Minimal evidence of understanding	2
					Adequate evidence of understanding	3
8. Process of randomisation (must refer to chance allocation)	0 Absent	1 Mostly unclear	2 Mostly clear	3 Very clear	Evidence of misunderstanding	0
					No evidence available	1
					Minimal evidence of understanding	2
					Adequate evidence of understanding	3

Total score	/24	/24

Comments arising during rating Section2 i

ii) Trial treatments

Topic	a. R information provision is:				b. P interaction suggests (tick box)	
9. Trial arm 1 processes	0<br>Absent	1<br>Mostly unclear	2<br>Mostly clear	3<br>Very clear	Evidence of misunderstanding	0
					No evidence available	1
					Minimal evidence of understanding	2
					Adequate evidence of understanding	3
10. Trial arm 1 costs or disadvantages	0<br>Absent	1<br>Mostly unclear	2<br>Mostly clear	3<br>Very clear	Evidence of misunderstanding	0
					No evidence available	1
					Minimal evidence of understanding	2
					Adequate evidence of understanding	3
11. Trial arm 1 benefits or advantages	0<br>Absent	1<br>Mostly unclear	2<br>Mostly clear	3<br>Very clear	Evidence of misunderstanding	0
					No evidence available	1
					Minimal evidence of understanding	2
					Adequate evidence of understanding	3
12. Trial arm 2 processes	0<br>Absent	1<br>Mostly unclear	2<br>Mostly clear	3<br>Very clear	Evidence of misunderstanding	0
					No evidence available	1
					Minimal evidence of understanding	2
					Adequate evidence of understanding	3
13. Trial arm 2 costs or disadvantages.	0<br>Absent	1<br>Mostly unclear	2<br>Mostly clear	3<br>Very clear	Evidence of misunderstanding	0
					No evidence available	1
					Minimal evidence of understanding	2
					Adequate evidence of understanding	3
14. Trial arm 2 benefits or advantages	0<br>Absent	1<br>Mostly unclear	2<br>Mostly clear	3<br>Very clear	Evidence of misunderstanding	0
					No evidence available	1
					Minimal evidence of understanding	2
					Adequate evidence of understanding	3
15. Trial arm 3 processes	0<br>Absent	1<br>Mostly unclear	2<br>Mostly clear	3<br>Very clear	Evidence of misunderstanding	0
					No evidence available	1
					Minimal evidence of understanding	2
					Adequate evidence of understanding	3
16. Trial arm 3 costs or disadvantages	0<br>Absent	1<br>Mostly unclear	2<br>Mostly clear	3<br>Very clear	Evidence of misunderstanding	0
					No evidence available	1
					Minimal evidence of understanding	2
					Adequate evidence of understanding	3

17. Trial arm 3 benefits or advantages	0 Absent · 1 Mostly unclear · 2 Mostly clear · 3 Very clear	Evidence of misunderstanding	0
		No evidence available	1
		Minimal evidence of understanding	2
		Evidence of understanding	3
Total score	/18 or /27	/18 or /27	
Comments Section 2ii			

Iii) Trial procedures

Topic	a. R information provision is:				b. P interaction suggests:	
18. Advantages or benefits of trial participation	0<br>Absent	1<br>Mostly unclear	2<br>Mostly clear	3<br>Very clear	Evidence of misunderstanding	0
					No evidence available	1
					Minimal evidence of understanding	2
					Adequate evidence of understanding	3
19. Costs / risks of trial participation.	0<br>Absent	1<br>Mostly unclear	2<br>Mostly clear	3<br>Very clear	Evidence of misunderstanding	0
					No evidence available	1
					Minimal evidence of understanding	2
					Adequate evidence of understanding	3
20. Option to refuse participation.	0<br>Absent	1<br>Mostly unclear	2<br>Mostly clear	3<br>Very clear	Evidence of misunderstanding	0
					No evidence available	1
					Minimal evidence of understanding	2
					Adequate evidence of understanding	3
21. Option to withdraw from participation.	0<br>Absent	1<br>Mostly unclear	2<br>Mostly clear	3<br>Very clear	Evidence of misunderstanding	0
					No evidence available	1
					Minimal evidence of understanding	2
					Adequate evidence of understanding	3
22. Options for further consultation to support decision making	0<br>Absent	1<br>Mostly unclear	2<br>Mostly clear	3<br>Very clear	Evidence of misunderstanding	0
					No evidence available	1
					Minimal evidence of understanding	2
					Adequate evidence of understanding	3
23. Outlines benefits (financial or otherwise) to professional or organisation of P participation.	0<br>Absent	1<br>Mostly unclear	2<br>Mostly clear	3<br>Very clear	Evidence of misunderstanding	0
					No evidence available	1
					Minimal evidence of understanding	2
					Adequate evidence of understanding	3
24. Outlines measures to protect confidentiality of participant data	0<br>Absent	1<br>Mostly unclear	2<br>Mostly clear	3<br>Very clear	Evidence of misunderstanding	0
					No evidence available	1
					Minimal evidence of understanding	2
					Adequate evidence of understanding	3
25. Outlines measures for compensation in case of adverse events	0<br>Absent	1<br>Mostly unclear	2<br>Mostly clear	3<br>Very clear	Evidence of misunderstanding	0
					No evidence available	1
					Minimal evidence of understanding	2
					Adequate evidence of understanding	3

Total score	/24	/24
**COmments on Part 2iii**		

Section 3: Global Judgements

Please respond to the following questions as applied up to the point of decision making about whether to accept the option to randomize or the end of the appointment. Please circle your response and add any comments below.

Does the recruiter consistently convey a position of equipoise and what evidence do you have to suggest this?

Yes Insufficient evidence No

Comments:

Do you believe the patient is in equipoise and what evidence do you have to suggest this?

Yes Insufficient evidence No

Comments:

Do you believe the patient accepts randomisation as a way to determine treatment and what evidence do you have to support this?

Yes Insufficient evidence No

Comments:

Do you believe that the patient is sufficiently informed by the end of the consultation to make an informed decision and what evidence to you have to support this?

Yes Insufficient evidence No

Comments:

Section 4 Ethnographic commentary of what occurs within the consultation.

There are no constraints on what can be included here. Feel free to add observations about **any elements of recruiter or patient contributions that stand out in this consultation** as regards **what** is discussed, **how** it is discussed and **how** it is understood.

Observations from this section will be used to develop the next version of the measure so please add comments on key issues that you feel need capturing and are not yet captured elsewhere. Shorthand notes are perfectly acceptable rather than written prose. It is intended that this section should be completed in around 10 minutes.
